# *Wolbachia w*AlbB inhibit dengue and Zika infection in the mosquito *Aedes aegypti* with an Australian background

**DOI:** 10.1371/journal.pntd.0010786

**Published:** 2022-10-13

**Authors:** Leon E. Hugo, Gordana Rašić, Andrew J. Maynard, Luke Ambrose, Catherine Liddington, Callum J. E. Thomas, Nisa Suraj Nath, Melissa Graham, Clay Winterford, B. M. C. Randika Wimalasiri-Yapa, Zhiyong Xi, Nigel W. Beebe, Gregor J. Devine

**Affiliations:** 1 QIMR Berghofer, Brisbane, Queensland, Australia; 2 School of Biological Sciences, University of Queensland, Brisbane, Australia; 3 CSIRO, Brisbane, Queensland, Australia; 4 Department of Medical Laboratory Sciences, The Open University of Sri Lanka, Colombo, Sri Lanka; 5 Department of Microbiology and Molecular Genetics, Michigan State University, East Lansing, Michigan, United States of America; University of Florida, UNITED STATES

## Abstract

Biological control of mosquito vectors using the endosymbiotic bacteria *Wolbachia* is an emerging strategy for the management of human arboviral diseases. We recently described the development of a strain of *Aedes aegypti* infected with the *Wolbachia* strain *w*AlbB (referred to as the *w*AlbB2-F4 strain) through simple backcrossing of wild type Australian mosquitoes with a *w*AlbB infected *Ae*. *aegypti* strain from the USA. Field releases of male *w*AlbB2-F4 mosquitoes resulted in the successful suppression of wild populations of mosquitoes in the trial sites by exploiting the strain’s *Wolbachia-*induced cytoplasmic incompatibility. We now demonstrate that the strain is resistant to infection by dengue and Zika viruses and is genetically similar to endemic Queensland populations. There was a fourfold reduction in the proportion of *w*AlbB2-F4 mosquitoes that became infected following a blood meal containing dengue 2 virus (16.7%) compared to wild type mosquitoes (69.2%) and a 6–7 fold reduction in the proportion of *w*AlbB2-F4 mosquitoes producing virus in saliva following a blood meal containing an epidemic strain of Zika virus (8.7% in comparison to 58.3% in wild type mosquitoes). Restriction-site Associated DNA (RAD) sequencing revealed that *w*AlbB2-F4 mosquitoes have > 98% Australian ancestry, confirming the successful introduction of the *w*AlbB2 infection into the Australian genomic background through backcrossing. Genotypic and phenotypic analyses showed the *w*AlbB2-F4 strain retains the insecticide susceptible phenotype and genotype of native Australian mosquitoes. We demonstrate that the *Wolbachia w*AlbB2-F4, in addition to being suitable for population suppression programs, can also be effective in population replacement programs given its inhibition of virus infection in mosquitoes. The ease at which a target mosquito population can be transfected with *w*AlbB2, while retaining the genotypes and phenotypes of the target population, shows the utility of this strain for controlling the *Ae*. *aegypti* mosquitoes and the pathogens they transmit.

## Author summary

Epidemics of arthopod-borne viral (arboviral) diseases affect millions of people and are becoming more frequent and widespread. A successful strategy for their control is the infection of mosquito populations with benign *Wolbachia* bacteria that render mosquitoes refractory to arbovirus infection. Here we show that a strain of the major disease vector *Ae*. *aegypti*, infected with *Wolbachia* following a simple back-cross mating procedure, is refractory to infection with dengue and Zika viruses. Importantly, the genetic background of the strain is equivalent to the native population, which is important for its persistence and competitive fitness, and for the regulatory approval of field releases.

## Introduction

Arthropod-borne viruses (arboviruses) transmitted by mosquitoes are responsible for global epidemics that are increasing in frequency and geographic scale [1]. Dengue is the most prevalent arboviral disease with 5.2 million cases reported to the WHO in 2019 [2]. The last three decades have seen the re-emergence of West Nile, Zika and chikungunya virus diseases in widespread outbreaks [3]. The majority of arboviruses lack effective vaccines with the exception of Japanese Encephalitis and Yellow Fever viruses. In most instances, mosquito management tools are the only options available for combating arbovirus transmission. In many regions, conventional insecticide-based campaigns are compromised by issues of coverage, insecticide resistance and cost, so alterative control tools are desperately required. In the last decade, the bacterium *Wolbachia pipientis* has emerged as a major tool for the management of mosquito populations and the pathogens they transmit [4].

*Wolbachia* are obligate intracellular endosymbiotic bacteria belonging to the order *Rickettsiales* that are widespread across arthropods because of their ability to manipulate the reproductive biology of their hosts [5]. *Wolbachia* are transmitted vertically, from female insects to their offspring and induce cytoplasmic incompatibility (CI), an effect where unviable offspring are produced when *Wolbachia*-carrying males mate with females without *Wolbachia* or with an incompatible *Wolbachia* strain. These traits facilitate *Wolbachia*’s ability to invade and spread. Stable, heritable *Wolbachia* infections in mosquitoes can result in phenotypes that have utility for mosquito population control. The CI phenotype can be used to “crash” local populations of mosquitoes through the mass release of *Wolbachia* infected male mosquitoes, a strategy referred to as “population suppression”. *Wolbachia* infections may also reduce the capacity of mosquitoes to transmit medically important arboviruses and parasites, a phenotype known as virus transmission blocking [6,7]. The primary mosquito vector of dengue, Zika and yellow fever viruses, *Aedes aegypti*, is not naturally infected with *Wolbachia*. Stable and heritable *Wolbachia* infections were initially established in *Ae*. *aegypti* through the microinjection of mosquito eggs [8–10]. Highly efficient virus transmission blocking has been demonstrated in mosquitoes infected with the *Wolbachia* strains *w*MelPop [6], *w*Mel [10], *w*Au [11] and *w*AlbB [12].

*Wolbachia-*induced virus transmission blocking can be harnessed by releasing *Wolbachia-*infected male and female mosquitoes in disease-endemic regions. A combination of maternal inheritance and CI drives the infection to spread slowly but irreversibly through a mosquito population, resulting in the replacement of the wild type with a more benign, disease refractory form [10,12–14]. This has been referred to as “population replacement” and various trials around the globe are evaluating the efficacy of population replacement for arbovirus disease control. Deployment of mosquitoes infected with *Wolbachia w*Mel have established the strain in local mosquito populations and led to significant reductions in dengue incidence, including 96% reduction in local dengue transmission in northern Australia [15,16], 73% reduction in dengue incidence in a quasi-experimental trial in a region of Yogyakarta, Indonesia [17] and 77.1% reduction in dengue incidence across Yogyakarta in a cluster randomised trial [18]. A quasi-experimental trial in Niterói, Brazil, observed a 69% reduction in dengue incidence, a 56% reduction in chikungunya incidence and a 37% reduction in Zika incidence [19].

The success of *Wolbachia* based population replacement relies on the persistence of the *Wolbachia* infection within local mosquito populations. *Wolbachia w*Mel has been detected at high prevalence for over eight years in north Queensland [15] and two years in Yogyakarta [17]. In other regions, the prevalence of *w*Mel has varied between release sites. The reductions in arbovirus prevalence in Niterói occurred when *w*Mel prevalence varied between 33 and 90% depending on the release zone [20]. Substantial decreases in *Wolbachia w*Mel infection prevalence were recently reported in a local population of *Ae*. *aegypti* in tropical central Vietnam [21]. Four years after their release, *Wolbachia w*Mel remained in just 5% of mosquitoes [21]. The most rapid of these declines correlated with the onset of the hot dry-season and the highest average and maximum weekly temperatures. High ambient temperatures typically associated with summer ‘heatwaves’ have caused reductions in the density of *w*Mel *Wolbachia* infections in mosquitoes in the laboratory [22,23] and reductions in prevalence and density following a north Queensland heat wave [24,25]. *Wolbachia* density is a critical factor defining CI [26], maternal inheritance and pathogen interference [27]. The susceptibility of the *w*Mel strain to heat stress has accelerated the search for alternative *Wolbachia* strains that demonstrate greater tolerance to heat while maintaining the pathogen blocking phenotype.

The *Wolbachia* strain *w*AlbB from the mosquito *Aedes albopictus* was the first to be stably transinfected into *Ae*. *aegypti*. The strain induced 100% maternal inheritance and strong CI in the new host [8]. In laboratory challenge assays, and in comparison with a non-*Wolbachia* infected strain, mosquitoes carrying *w*AlbB were infected with dengue at a lower rate, the titres of dengue virus in infected bodies were reduced and the proportion of mosquitoes with dengue virus in saliva (those potentially capable of transmission) was reduced by ≥ 37.5% [28]. The *w*AlbB strain reduced dengue transmission potential of mosquitoes to a greater extent than *w*Mel in a robust side-by-side comparison [29]. *Wolbachia w*AlbB also appears more robust to heat stress than the *w*Mel strain [23]. Population replacement trials using the *w*AlbB strain are rare, but in 2017, releases at six trial locations in Selangor, Malaysia, resulted in rapid establishment of *Wolbachia* [30] and *Wolbachia* infection densities and tissue distributions remained unchanged in mosquitoes after 20 months [12]. Field collected mosquitoes also demonstrated strong dengue inhibition in the laboratory. The *w*AlbB strain has clear utility for population replacement, particularly in tropical climates.

We recently described the successful suppression of a population of *Ae*. *aegypti* in northern Australia following mass releases of male mosquitoes from an Australian strain of *Ae. aegypti* infected with *Wolbachia w*AlbB [25]. This mosquito strain, referred to as *w*AlbB2-F4, was established by a four-generation backcross that mated male *Wolbachia*-free mosquitoes from northern Australia with females from an imported *w*AlbB infected *Ae*. *aegypti* strain (WB2). WB2 is an *Ae*. *aegypti* strain from the USA infected with *Ae*. *albopictus* B lineage *Wolbachia* established by ZX. The *w*AlbB2-F4 strain, which is the subject of the current study, displayed complete maternal inheritance of *Wolbachia* and cytoplasmic incompatibility with wild type mosquitoes from Queensland Australia and mosquitoes infected with the *w*Mel strain of *Wolbachia*. Mass releases of male *w*AlbB2-F4 led to ratios of over 10 infected males per wild male and population suppression of >80% [25]. Here we demonstrate that the *w*AlbB2-F4 strain is also suitable for mosquito population replacement strategies aiming to reduce flavivirus transmission. *w*AlbB2-F4 females demonstrated strong resistance to infection with dengue 2 virus (DENV-2) and Zika virus (ZIKV) and are equivalent to north Queensland mosquitoes genotypically. Proof of genetic similarity was required because regulatory approval for release was contingent on limiting the introduction of alien genetic material and ensuring that the phenotype reflected the inherent insecticide susceptibility of Australian *Ae*. *aegypti* populations.

## Results

### *Wolbachia w*AlbB is distributed throughout the mosquito but infection density varies between tissues

**Fig 1:**
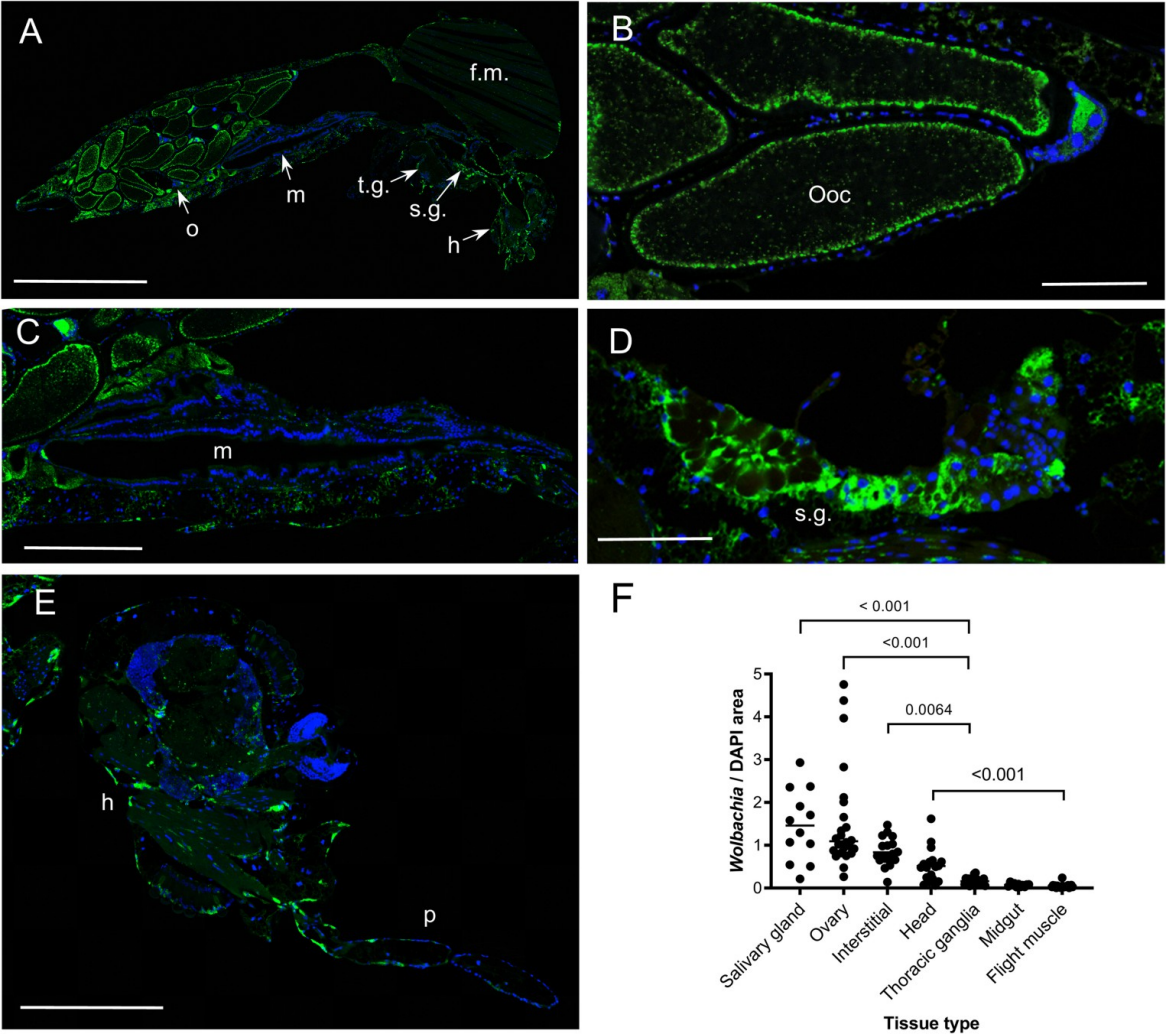
Histology of *Wolbachia* infection in the *Aedes aegypti w*AlbB2-F4 strain. *Wolbachia* infection was observed across mosquito organs/tissue types by immunofluorescence analysis (IFA) using a rabbit polyclonal antibody against the *Wolbachia* surface protein (WSP) as the primary antibody and Alexa Fluor 488-conjugated donkey anti-rabbit antibody as the secondary antibody. DNA was stained using DAPI. (A) Example of whole body section showing IFA staining. (B-E) High resolution images of *Wolbachia* staining in oocytes, midgut, salivary gland and heads, respectively. (F) Quantification of *Wolbachia* staining density. Staining areas were quantified by image analysis and expressed as a ratio of *Wolbachia* staining over DAPI staining for each organ/tissue. The median staining densities differed significantly between groups (Kruskal-Wallis statistic = 95.37, N = 124). *P* values are reported for comparisons where medians differed significantly by Dunn’s multiple comparison test (α = 0.05, 21 comparisons). Green, *Wolbachia*,. Blue DNA. h, head. f.m., flight muscles. m, midgut. o, ovary. ooc, oocyte. p, proboscis. s.g., salivary glands. t.g., thoracic ganglia. Scale bars: A: 1.00 mm, B, D: 0.10 mm. C, E: 0.25 mm.

The density of *Wolbachia* within female *w*AlbB2-F4 mosquitoes was determined by immunofluorescence analysis (IFA) using an antibody against the *Wolbachia* surface protein (WSP) and DAPI staining for DNA (Fig 1). *Wolbachia* infection was widespread though internal mosquito tissues but the staining density (determined from the ratio of the area of *Wolbachia* WSP staining to mosquito cellular DNA) varied between tissues (Fig 1F and S1 Dataset). Of several tissue types examined, median *Wolbachia* staining density was highest in salivary glands (Fig 1D and 1F), followed by ovaries (Fig 1A, 1B and 1F). Interstitial spaces and heads had moderate staining densities (Fig 1E and 1F). *Wolbachia* staining densities were significantly higher in salivary glands, ovaries and interstitial spaces than in thoracic ganglia, midgut and flight muscle tissue (Kruskal-Wallis tests; Fig 1F)).

### The *w*AlbB2-F4 strain is resistant to dengue and Zika viruses

We assessed the level of *Wolbachia-*induced suppression of DENV-2 and ZIKV in *w*AlbB2-F4 mosquitoes compared to Australian wild type (*Wolbachia*-free) *Ae*. *aegypti*. Both wild type and *w*AlbB2-F4 mosquitoes were fed a blood meal containing a contemporary strain of DENV-2 before incubation under insectary conditions.

**Fig 2:**
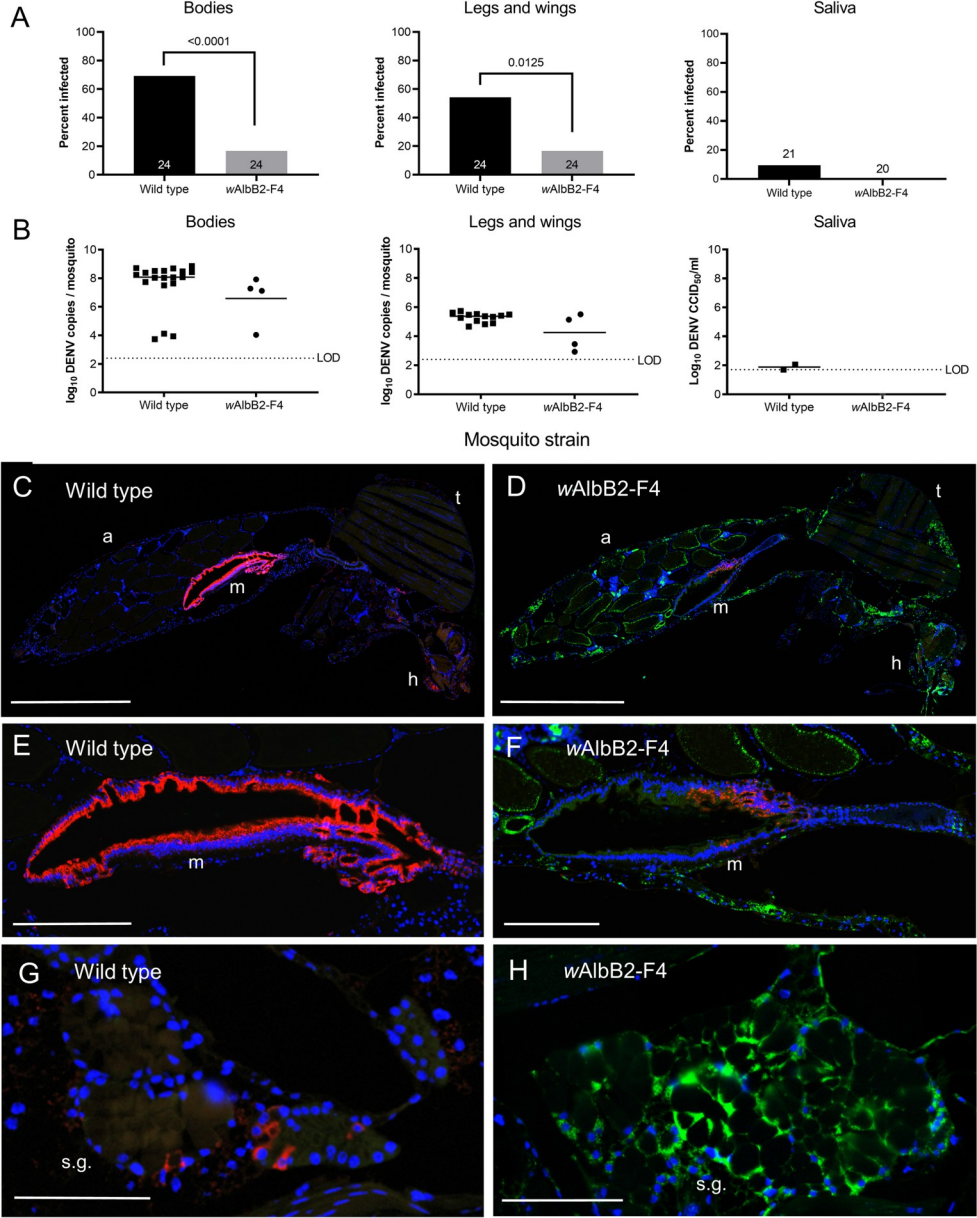
*Wolbachia* inhibit dengue 2 infections in *Ae*. *aegypti w*AlbB2-F4 mosquitoes. (A) DENV-2 virus prevalence in the bodies, legs and wings, and saliva of *Ae*. *aegypti w*AlbB2-F4 and wild type mosquitoes 14 d after feeding on a blood meal containing 1 × 10^6.6^ CCID_50_/ml (in C6/36 cells) of DENV-2 virus. *P* values are included for comparisons with significant differences in mosquito infection rates between *w*AlbB2-F4 and wild type mosquitoes (Fisher’s Exact test). (B) DENV-2 infection intensity in mosquitoes in A. Virus copy numbers were determined from bodies, legs and wings and saliva samples using quantitative reverse-transcriptase PCR (qRT-PCR). *P* values are shown for comparisons with significant different median virus copy numbers between *w*AlbB2-F4 and wild type mosquitoes (Mann-Whitney test). (C) Example whole body midsagittal section from a wild type mosquito dual stained for *Wolbachia* (green) and DENV-2 (red). (D) Whole body section of a *w*AlbB2-F4 female showing restriction of virus to the midgut. (E-F) High resolution images of midguts from wild type and *w*AlbB2-F4 mosquitoes showing lower DENV-2 staining density in the *w*AlbB2-F4 midgut. (G-H) High resolution images of salivary glands from wild type and *w*AlbB2-F4 mosquitoes showing dense *Wolbachia* infection and absence of DENV-2 infection in the latter. a, abdomen. h, head. LOD, limit of detection. m, midgut. s.g., salivary glands. t, thorax. Scale bars: C-D, 1 mm. E-F, 0.25 mm, G-H: 0.10 mm.

The presence and intensity of DENV infection in mosquito tissues was analysed by quantitative reverse transcriptase PCR (qRT-PCR) targeting a region of the DENV 3’ untranslated region (UTR) [27]. At 14 d post feeding (dpf), 69.2% of wild type mosquitoes were infected with DENV, whereas only 16.7% of *w*AlbB2-F4 mosquitoes were infected, representing a highly significant reduction (Fisher’s Exact test, *P* < 0.001, Fig 2A). Furthermore, there was a significant reduction in the proportion of mosquitoes with virus in legs and wings (Fisher’s Exact test, *P* = 0.0145, Fig 2A). The median number of virus copies in infected mosquito bodies and legs and wings samples did not differ significantly between *w*AlbB2-F4 and WT mosquitoes (Mann-Whitney tests, Fig 2B), however the tests were restricted by the low number of infected individuals in the *w*AlbB2-F4 strain. The presence and quantity of live virus in saliva was analysed by cell culture ELISA [31]. DENV was not detected in the saliva of *w*AlbB2-F4 mosquitoes, but it was detected in the saliva of 9.5% of wild type mosquitoes (Fig 2A). The relative infection density of *Wolbachia* and DENV was visualised using dual-antibody IFA, using the *Wolbachia* WSP antibody, an antibody against flavivirus non-structural protein 1 (NS1) and DAPI staining. DENV infection could be observed in tissues throughout the head, thorax and abdomen of wild type mosquitoes (Fig 2C). In contrast, of the *w*AlbB2-F4 mosquitoes that were infected, virus was restricted to the midgut (Fig 2D). Staining density appeared higher in the midguts of wild type mosquitoes (Fig 2E) compared with *w*AlbB2-F4 mosquitoes (Fig 2F). DENV was observed in the salivary glands of wild type mosquitoes, however it could not be detected from salivary glands of *w*AlbB2-F4 mosquitoes (Fig 2F). In *w*AlbB2-F4 females the restriction of DENV infection to the midgut corresponded with a low density of *Wolbachia* infection in that tissue (Figs 2F and 1F).

**Fig 3:**
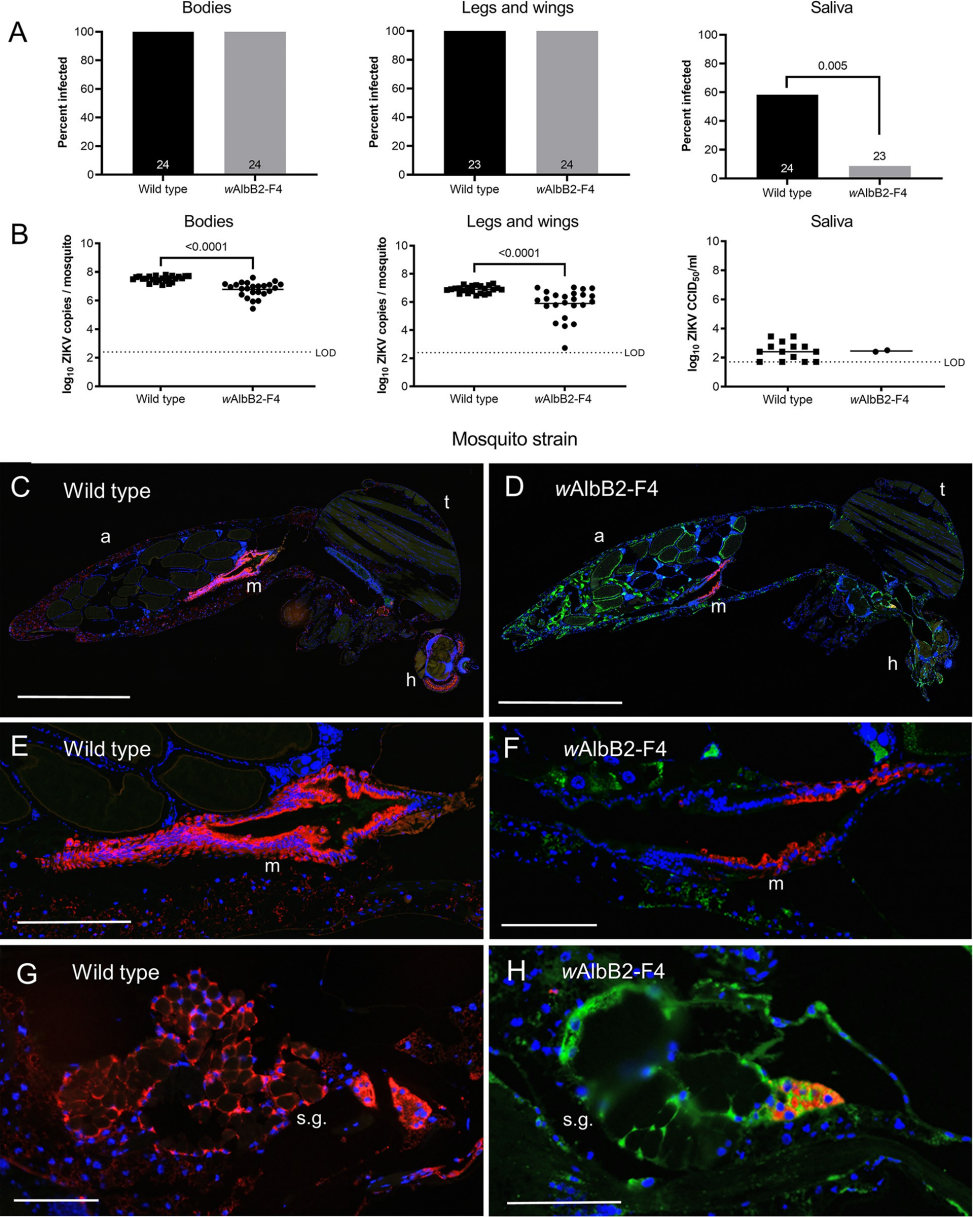
*Wolbachia* inhibit ZIKV infections in *Ae*. *aegypti w*AlbB2-F4 mosquitoes. (A) ZIKV virus infection prevalence in *Ae*. *aegypti* wild type and *w*AlbB2-F4 mosquitoes 14 d after feeding on a blood meal containing 1 × 10^8.5^ CCID_50_/ml (in C6/36 cells) of Zika virus. *P* values are included for comparisons with significant differences in mosquito infection rates between *w*AlbB2-F4 and wild type mosquitoes (Fisher’s Exact test). (B). ZIKV infection densities in mosquitoes from A. Virus copy numbers were determined from bodies, legs and wings and saliva samples using qRT-PCR. *P* values are shown for comparisons with significant different median virus copy numbers between *w*AlbB2-F4 and wild type mosquitoes (Mann-Whitney test). (C-D) Example whole body midsagittal sections of wild type and *w*AlbB2-F4 mosquitoes dual stained for *Wolbachia* (green) and ZIKV (red). (E-F) High resolution images of midguts from wild type and *w*AlbB2-F4 mosquitoes, respectively, showing relatively lower staining density for *w*AlbB2-F4 mosquitoes. (G-H) High resolution images of midguts from wild type and *w*AlbB2-F4 mosquitoes, respectively. Infection was limited and spatially restricted in *w*AlbB2-F4 mosquitoes. a, abdomen. h, head. LOD, limit of detection. m, midgut. s.g. salivary gland. t, thorax. Scale bars: C-D, 1 mm. E-F, 0.25 mm, G-H: 0.10 mm.

Mosquitoes from the wild type and *w*AlbB2-F4 mosquitoes were also provided a blood meal containing a strain of Zika virus isolated from a febrile patient in Paraiba State during the 2015/2016 Brazil epidemic (Fig 3A). Mosquitoes were incubated for 14 dpi and the presence and intensity of Zika virus infection in mosquito segments was analysed by qRT-PCR [32]. All wild type and *w*AlbB2-F4 mosquitoes had detectable virus in bodies and legs and wings at 14 d post feeding (Fig 3A). However, significantly fewer virus copy numbers were observed in both the bodies (Mann Whitney U = 31, *P* < 0.001) and legs and wing tissue (Mann Whitney U = 71.5, *P* < 0.001) of the *w*AlbB2-F4 mosquitoes at 14 dpi compared to wild type mosquitoes (Fig 3B). Live ZIKV was detected and quantified in mosquito saliva using the NS1 antibody as described above. Importantly, there was a highly significant reduction (Fisher’s Exact test, *P* = 0.005) in the proportion of *w*AlbB2-F4 mosquitoes that expectorated virus in saliva compare to wild type mosquitoes.

Dual antibody IFA of ZIKV using the anti-WSP and flavivirus NS1 antibody (as above) revealed that, by 14 dpf, ZIKV had disseminated widely in mosquitoes from the wild type strain (Fig 3C) but was generally restricted to midgut tissue in females from the *w*AlbB2-F4 strain (Fig 3D). ZIKV infection in midgut tissue reached high densities in wild type females (Fig 3E) but was visibly lower in the midgut tissue of *w*AlbB2-F4 females. Similarly, the salivary glands of wild type females were observed to have dense ZIKV infection, but the infection appeared lower and spatially restricted in *w*AlbB2-F4 salivary glands (Fig 3F). In *w*AlbB2-F4 females, the highest densities of ZIKV infection were therefore observed in midgut tissue, in which the *Wolbachia* infection density was lowest (Figs 3F and 1F).

### *w*AlbB2-F4 strain has the genomic background of Australian *Aedes aegypti* and is susceptible to commonly-used insecticides

**Fig 4:**
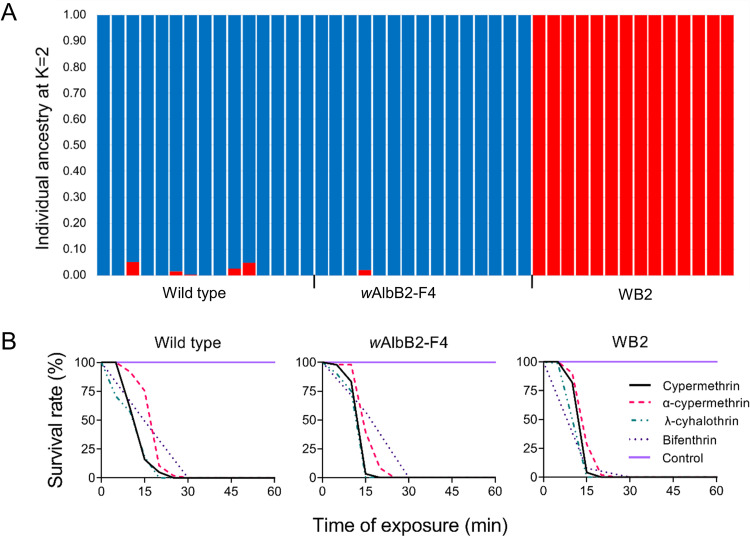
Analysis of genetic equivalency of *w*AlbB2-F4 strain to Australian *Aedes aegypti*. (A) All mosquitoes from the *w*AlbB2-F4 strain have >98% ancestry (Q-values) from the Australian wild-type strain (and not the WB2 strain), indicating a successful backcrossing procedure. (B) The *Wolbachia* infected *Aedes aegypti w*AlbB2-F4 strain has equivalent insecticide susceptibility to the parental Queensland wild type mosquitoes and *Ae*. *aegypti w*AlbB2 strains. Survival rates of mosquitoes were recorded following exposure to diagnostic doses of alpha-cypermethrin, cypermethrin and lambda-cyhalothrin using CDC bottle bioassays. Survival rates following exposure to a diagnostic dose of bifenthrin were determined using WHO filter paper assays.

Double digest RAD genome-wide sequencing of mosquitoes from the *w*AlbB2-F4 strain and the two strains used for its generation (WB2 strain and a wild type Australian strain) revealed that the backcrossing procedure resulted in *w*AlbB2-F4 mosquitoes that shared > 98% of the wild-type genome (Fig 4A and S2 Dataset). Q values from the ADMIXTURE analysis show this ancestry percentage is consistent across all analysed *w*AlbB2-F4 individuals (Fig 4A).

Females from the *w*AlbB2-F4 strain and the parental wild type and WB2 strains were fully susceptible to the insecticides cypermethrin, α-cypermethrin, λ-cyhalothrin and bendiocarb (Fig 4B). All females (n = 40–82) died following exposure to the diagnostic doses of these insecticides for 30 mins.

We also tested for the presence of pyrethroid resistance genotypes in these strains. The *kdr* mutations, V410L, V1016I and F1534C were not detected in the *w*AlbB2-F4 strain, nor the two parent strains, WB2 and wild type, whereas the respective wild type alleles were amplified in 100% of qPCR reactions from these strains. As a positive control, these *kdr* mutations were detected from >47% of mosquitoes from a reference strain from Mexico.

## Discussion

We recently reported the generation of an Australian colony of *Ae*. *aegypti* infected with the *w*AlbB2 strain of *Wolbachia*. This was used to suppress native mosquito populations in trial sites in northern Australia [25]. Here we show that the strain is also likely to be amenable for use in mosquito population replacement interventions; whereby persistent *Wolbachia* infections are established in target populations of mosquitoes to substantially reduce their capacity for transmitting pathogenic arboviruses [10,14]. Compared to wild type (*Wolbachia*-free) mosquitoes, 76% fewer *Ae*. *aegypti* from this (*w*AlbB2-F4) strain became infected following a blood meal that contained a contemporary DENV-2 virus. Significantly fewer *w*AlbB2-F4 mosquitoes developed a disseminated infection and virus could not be detected in saliva expectorates. Females from the *w*AlbB2-4 and wild type strains were susceptible to infection from a high dose of Zika virus, but 85% fewer *w*AlbB-2 had virus in their saliva (a proxy for transmission potential) when compared to the wild type. We showed that the *w*AlbB infection follows a typical pattern through different tissues, including high density in oocytes, and that increased flavivirus density in midgut tissue is associated with low tissue density of *Wolbachia* infection. We also showed that the *Ae*. *aegypti w*AlbB2-F4 strain has an equivalent genetic background to wild mosquitoes from Queensland, Australia, having >98% similarity to the native mosquitoes as estimated from genome-wide SNP markers. Susceptibility to the pyrethroid insecticides used for *Ae. aegypti* control in Queensland was preserved in our *w*AlbB2-F4 strain. Maintenance of the native genotype may be critical for the successful invasion of *Wolbachia* into wild mosquito populations [33] and was a prerequisite for regulatory approval in Australia which has strong biosecurity controls.

There was a four-fold reduction in the proportion of *Ae*. *aegypti w*AlbB2-F4 mosquitoes that became infected with a contemporary DENV-2 strain (16.7%) compared to wild type mosquitoes (69.2%) and a three-fold reduction in the proportion of mosquitoes that developed a disseminated infection (16.6% in *w*Ablb2-F4 against 54.2% in wild-type). The reduced susceptibility of *w*AlbB2-F4 mosquitoes to infection and dissemination of DENV correlates well with results from an independently-generated *Ae*. *aegypti* strain transinfected with *w*AlbB *Wolbachia* (referred to as the *Ae*. *aegypti* WB1 strain) [28] and for *w*AlbB introgressed into a Taiwanese *Ae*. *aegypti* background in which all four DENV serotypes were inhibited [34]. We were also able to reveal differences in tissue distribution between *Wolbachia* and DENV-2 infection by performing dual antibody immunofluorescence analysis. Infections in wild type mosquitoes at 14 d post feeding were characterized by intense staining in midguts and isolated pockets of disseminated infection throughout the body. Conversely, in *w*AlbB2-F4, DENV-2 infection was restricted to midgut tissue, which had the lowest tissue densities of *Wolbachia* infection, in keeping with the reported positive relationship between *Wolbachia* density and virus inhibition for most *Wolbachia*-transfected mosquitoes [35]. As the mosquito midgut is the first tissue to encounter arbovirus from an infected blood meal, the establishment of virus infection in these mosquitoes is probably due to the absence of *Wolbachia* in midgut tissue, whereas the inhibition of virus dissemination is the result of widespread *Wolbachia* infection in surrounding tissues.

For a high dose of an epidemic strain of Zika virus all mosquitoes from *w*AlbB-F4 and wild type strains developed primary and disseminated infections. However, there was a highly significant reduction in the proportion of *w*AlbB2-F4 mosquitoes producing virus in saliva (8.7% compared to 58.3% for wild type). The infection of all tested mosquitoes was likely due to the very high titre of Zika virus fed to mosquitoes (10^8.5^ 50% cell culture infectious dose per ml [CCID_50_/ml] in C6/36 cells). The antiviral effect of *Wolbachia* infection was evident from the significantly lower ZIKV loads in bodies and legs and wings of *w*AlbB2-F4 mosquitoes, and the substantially lower proportion of *w*AlbB2-F4 mosquitoes that produced detectable ZIKV in saliva. The presence of virus in saliva is a proxy for the ability of a mosquito to transmit the virus and is therefore the most epidemiologically relevant measure. Our results correlate closely to results from similar experiments in other *Ae*. *aegypti* transinfected with the *Wolbachia w*AlbA [36] and *w*AlbB [11] strains. For both strains, *Wobachia* infection was associated with modest reductions to mosquito infection and dissemination rates but induced complete blockage of virus transmission (no virus was detected from salivas). Their results were also supported by our dual immunofluorescence analysis of ZIKV and *Wolbachia*. DENV and ZIKV was generally restricted to midgut tissue in the *w*AlbB2-F4 strain.

We confirmed that the *w*AlbB2-F4 strain is genetically equivalent to wild type *Ae*. *aegypti* from Queensland. For the maximally-divergent parental strains, the backcross mating strategy used for the creation of *w*AlbB2-F4 should, arithmetically, transfer >90% of the Australian genome [37] to the new strain. In fact, we achieved a > 98% match. Maintaining the genetic background of released mosquitoes may be critical for the success of mosquito population replacement interventions [38]. It may increase the likelihood that the released mosquitoes will survive and mate competitively within the target population, providing the best chance for persistence of *Wolbachia* infection and therefore the virus blocking phenotype. It is particularly important that released mosquitoes have equivalent insecticide susceptibility to the target population. Insecticides remain the primary means of mosquito control and various genetically determined mechanisms of insecticide resistance have evolved within *Ae*. *aegypti* populations. Releases of *w*MelBr-infected *Ae*. *aegypti* failed to achieve population replacement in Rio de Janeiro due to an absence of insecticide resistance in this strain in an environment where there is high household use of insecticides [33]. These conditions had selected for resistance in the resident population. Once the infected strain was backcrossed with resistant mosquitoes from Rio de Janeiro (*w*MelRio), the replacement intervention was successful [33]. Genetic equivalency between the released and target mosquitoes is also important in particular circumstances where the field-release of any potentially beneficial organism is strictly regulated. One of the conditions of field release for our strain was to demonstrate that the native genotype had been conserved [25]. We have provided a rare genetic proof that introgression of a *w*AlbB2 *Wolbachia* infection into new *Ae*. *aegypti* strains can be achieved by a straightforward process of back crossing that conserves the genotype of the target population [34].

A pertinent question is whether *Wolbachia* strains other than the widely distributed *w*Mel strain are necessary for future population replacement interventions. Recent laboratory and field evidence indicates that *Wolbachia* infections can be susceptible to heat stress, causing decreased *Wolbachia* infection density after larvae are exposed to extreme climatic temperatures [22–24]. Decreases in *w*Mel *Wolbachia* infection density in mosquitoes due to heat wave conditions experienced in north Queensland were transient [22, 24] and have not prevented the long term persistence of the strain in this region. Similarly, the *w*Mel strain has persisted in *Ae*. *aegypti* populations in Yogyakarta, Indonesia, where it is associated with a 77% reduction in human dengue prevalence [18]. *w*Mel establishment has been more variable elsewhere; prevalence in *Ae*. *aegypti* was between 33–90% in release sites in Niterói, Brazil. High temperatures may have contributed to the heterogeneity in this study [19] and the low prevalence of *w*Mel in Nha Trang, Vietnam [21]. Side by side comparisons show the *w*AlbB strain to be more robust to heat stress than the *w*Mel strain [23, 39], however the utility of a *Wolbachia* strain is also determined by factors including the competitiveness of infected mosquitoes with uninfected wild mosquitoes and persistence of *Wolbachia* infections in the mosquito eggs. Characterisation of *Wolbachia* strains with varying traits, including thermal tolerance, could allow for the optimisation of *Wolbachia* strategies that are tailored to a particular DENV endemic region and to mitigate potential effects of increasing global temperatures [40].

This study has limitations. A low percentage of wild type mosquitoes infected with DENV-2 developed a salivary gland infection, precluding statistical comparison of the effects of *Wolbachia* infection in this tissue. This may be due to factors determining the compatibility of mosquito and virus strains that restricted the susceptibility of salivary glands, the blood meal titre being too low or an undetermined technical factor. However, the highly significant reduction in DENV-2 prevalence in mosquito bodies due to *Wolbachia* would likely have resulted in a significant reduction in DENV prevalence in salivary gland tissue if mosquito and virus were perfectly compatible. Vector competence was assessed in single experiments for dengue and Zika virus, on single strains for each virus. However, the similar trend in *Wolbachia* effects on two flaviviruses in this study and a growing body of evidence of virus blocking of the *Wolbachia w*AlbB strain in other mosquito genetic backgrounds [28,34,41] provide strong evidence of the ability of this strain to inhibit pathogenic flavivirus infection in mosquitoes.

The *Wolbachia w*AlbB strain has potential to be applied in mosquito population replacement interventions targeting flavivirus diseases. Traits demonstrated here and elsewhere include; substantial reductions to flavivirus vector competence of transinfected mosquitoes [36,41], induction of complete maternal inheritance and cytoplasmic incompatibility [25], ease of introgression into target mosquito genetic backgrounds and tolerance of heat stress [11,23]. Future studies should assess competitiveness against *Wolbachia*-uninfected mosquitoes from the same genetic background.

## Methods

### Mosquito strains

Wild type *Ae*. *aegypti* were established from egg collections made in Cairns and Innisfail in 2015 and 2016, respectively, and they were confirmed to be uninfected with *Wolbachia* by PCR. Establishment of the *Ae*. *aegypti w*AlbB2-F4 strain was described in [25]. Briefly, matings were set up with one male from the wild type Australian strain and three virgin females from the USA WB2 strain, imported from the Michigan State University. These females were then blood fed and allowed to lay eggs. The F1 eggs were hatched, female pupae separated and reared till adulthood to be then mated with wild type males. The procedure was repeated for two additional generations to obtain the F4 generation of the backcross refer to as the *w*AlbB2-F4 strain. The mosquito colonies were maintained in the QIMR Berghofer insectary at 28°C, 70% relative humidity and 12:12 hr light cycling with dawn and dusk fading. Adults were maintained in 30 × 30 × 30 cm cages (BugDorm, MegaView Science Education Services Co., Ltd., Taiwan) and provided with 10% sucrose solution *ad libitum* and defibrinated sheep blood (Serum Australis, Manila, NSW, Australia) once per week. To provide mosquitoes for experiments, eggs were flooded in plastic trays and larvae were maintained at a density of 500 larvae in 4 L of aged tap water and fed ground TetraMin tropical fish food flakes *ad libitum* daily before the resulting pupae were sorted into adult emergence trays and transferred to cages.

### Viruses

A strain of DENV-2 (QML16) originally isolated from a dengue fever patient in Australia in 2015 was provided by Prof John Aaskov, Queensland University of Technology, Australia. A strain of ZIKV (KU365780) isolated from a Zika virus disease patient in Joao Pessoa, Paraiba State, Brazil on 18 May 2015 was provided by Pedro Fernando da Costa Vasconcelos, Evandro Chagas Institute, Brazil. Both viruses were propagated in C6/36 cells at 28°C, 5% CO_2_ for 5 d. Infected cell culture supernatants were harvested for these experiments. ZIKV was concentrated using an Amicon Ultra-15 Centrifugal Filter Unit with an Ultracel-100 membrane (Merck Millipore, Darmstadt, Germany).

### Vector competence of the *Ae*. *aegypti w*AlbB2-F4 strain for dengue and Zika viruses

#### Mosquito infection

Approximately 100 *w*AlbB2-F4 or wild type females were placed in 750 ml plastic containers with gauze lids and fed mixtures of DENV-2 or Zika virus in defibrinated sheep blood (Serum Australis) using glass artificial membrane feeders [42]. Blood virus mixtures consisted of DENV-2 supernatant and defibrinated sheep blood at a ratio of 1:1, or ZIKV stock and defibrinated sheep blood at a ratio of 1:5 and samples of the blood virus mixtures were taken before and after the feeding period to determine virus titres. The average titre fed was determined to be 1 × 10^6.6^ and 1 × 10^8.5^ CCID_50_/ml in C6/36 cells for DENV-2 and ZIKV, respectively. After the feeding opportunity, all mosquitoes were anaesthetised with CO_2_ and placed on a petri dish on ice. Non- or partially engorged mosquitoes were discarded and fully engorged mosquitoes were placed in containers, provided with 10% sugar solution *ad libitum* and housed in an environmental chamber (Panasonic, Osaka, Japan) set at 28°C, 75% RH, and lighting conditions described above. Mosquitoes were harvested 14 d after blood feeding, anaesthetized and placed on ice. Legs and wings were removed and placed into 2 ml screw cap vials with three 2.3 mm zirconium silica glass beads. Saliva was collected by placing mosquito bodies on double sided tape and positioning a 200 ml pipette tip containing 10 μl of saliva collection fluid (10% FBA, 10% sugar [43]) over the proboscis of each mosquito for 20 min. The contents were expelled into a 1.5 ml microfuge tube. Each body was placed into a 2 ml screw-cap tube with beads as described above.

#### Quantification of the virus from mosquito bodies

Virus nucleic acid was extracted using the Roche High Pure virus nucleic acid extraction kit by adding 200 μl of the working binding buffer to the tubes containing bodies or legs & wings and homogenizing the tissues by shaking the tubes for 1 min 30 s using a Mini Beadbeater-96 (BioSpec Products, Bartlesville, OK, USA). The tubes were centrifuged for 8,000 × g for 1 min. 50 μl Proteinase K was added and the procedure was continued as described in the manufacturer’s protocol.

Dengue virus quantification was performed by One-step RT-qPCR using the Taqman Fast Virus 1-Step Master Mix and primers and probe targeting the DENV 3’UTR region described by Frentiu et al [27]. Ten μl reactions contained 2.5 μl of 4 x Taqman Fast virus mastermix, 400 nM of each primer, 250 nM of probe and 1 μl of virus nucleic acid extraction. Primers and probe were synthesized by Macrogen (Macrogen, Seoul, Korea). Thermal cycling was performed using a Corbett Rotorgene 6000 (QIAGEN/Corbett, Sydney, NSW, Australia) with incubation at 50°C for 5 min, 95°C for 20 s, then 40 cycles of 95°C for 3 s and 60°C for 30 s. Absolute quantification of virus copy number was performed using the Rotorgene 6000 software package using a standard curve derived from 10-fold serial dilutions of a linearized plasmid containing the 3’UTR gene [27]. Zika virus quantification was performed by One-step RT-qPCR using the reaction mix and thermocycling conditions described above but with primers ZIKV 911C and ZIKV 835 and probe ZIKV 860-FAM [32]. A 10-fold serial dilution series of a linearized plasmid containing the target sequence [31] was used for determination of Zika virus copy number.

#### Cell culture ELISA for determination of live virus in blood meals and mosquito saliva

Blood virus mixtures were titrated by ten-fold serial dilution in virus media (RPMI 1640 cell culture media supplemented with 5% fetal bovine serum [FBS] and 1% Penicillin-Streptomycin [Gibco]) in a 96-well plate before transferring dilutions to equivalent wells of a 96 well plate containing near confluent C6/36 cell monolayers. The inoculated cells were incubated at 28°C, 5% CO_2_, for five d. Five-fold dilutions of saliva samples were tested and the inoculated cells were incubated for six d. After incubation, cell monolayers were fixed by adding 100 μl of ice cold 80% acetone / PBS and incubating plates at -20°C for 1 hr. The acetone was removed and plates were rinsed three times in PBS. DENV and Zika antigen was detected by performing an Enzyme Linked Immunosorbant Assay (ELISA) targeting *Flavivirus* NS1 protein. Fixed cells were blocked in 100 μl of blocking buffer (1% [w/v] bovine serum albumin in PBS) at room temperature for 1 hr. Cells were then washed three times in PBS/0.05% Tween 20 (PBS-Tween). The cells were incubated with 50 μl 4G4 anti-Flavivirus NS1 monoclonal hybridoma supernatant [44] (1:40 in PBS-Tween) and then washed three times in PBS-Tween. Cells were incubated with 50 μl Horse Radish Peroxidase (HRP-) conjugated goat anti-mouse antibody (Dako) (1:2000 in PBS-Tween) before being washed four times in PBS-Tween. Plates were dried and wells were incubated with 50 μl of Tetramethylbenzidine (TMB) Liquid Substrate for Membranes (Sigma Aldrich) for 30 min at room temperature. Blue staining of the cell monolayer indicated the presence of virus infection in cells. The 50% Cell Culture Infectious Dose (CCID_50_) per ml in C6/36 cells was calculated using the method of Reed and Muench [45].

### Histological analysis of *Wolbachia* and flavivirus infections

Infections of *Wolbachia* and DENV-2 or ZIKV were detected in thin paraffin sections from mosquitoes by immunofluorescence analysis based on established protocols [31] with the following modifications for dual staining of *Wolbachia* and flaviviruses. Nonspecific antibody binding by incubating mosquito sections in 10% donkey serum for 60 min. Excess serum was decanted and the first primary antibody, 4G4 mouse anti-Flavivirus NS1 [44], was applied undiluted overnight in a humidified chamber. Sections were washed three times in Tris buffered saline plus 0.025% Tween 20 (TBS_TW_).10% donkey serum was applied for 15 min before a rabbit anti-WSP polyclonal antibody diluted 1:500 [35] in 10% donkey serum was applied for 2 h at room temperature in a humidified chamber. Sections were washed three times in TBS_TW_. Alexa Fluor donkey anti-mouse 555 diluted 1:300 and Alexa Fluor 488 donkey anti-rabbit diluted 1:1000 in TBS was applied for 60 min. Sections were washed three times in TBS_T_w. Sections were counterstained with DAPI for 10 min, washed several times in PBS and mounted with Vector Vectashield or Dako Fluorescence Mount. Microscopy was performed using an Aperio ScanScope fluorescent microscope using filters for DAPI, Alexa 555 (Cy 3) and Alexa 488 (FITC) and exposure times of 0.1 s, 0.2 s and 0.16 s, respectively. The relative staining density of *Wolbachia* to DAPI stained DNA was measured digitally from images using established protocols [31].

### Genome-wide characterization of the *w*AlbB2-F4 and parental strains

#### DNA extraction, sequencing, and genotyping of individual mosquitoes

Total genomic DNA was extracted from individual mosquitoes using DNeasy Blood and Tissue DNA extraction kit (Qiagen, Hilden, Germany) according to the manufacturer’s instructions. We prepared one double-digest RADseq library with DNA from 44 individually-barcoded mosquitoes: 15 from the *w*AlbB2-F4 strain, 15 from the wild-type Australian strain (Cairns), and 14 mosquitoes from the WB2 strain. The library was prepared following the protocol described in Rašić et al. [46], and sequenced on one lane of the Illumina HiSeq4000 platform. The sequencing data were demultiplexed and processed (trimmed to 90 bp and filtered for quality) using the bash script/pipeline from Rašić et al. [46]. The high-quality reads were aligned to the *Ae*. *aegypti* genome assembly version AaegL5 [47] with the aligner Bowtie [48]. Unambiguously mapped reads were converted to the bam format and processed in SAMtools [49]. The sorted bam files were passed to the ANGSD pipeline, where the SAMtools algorithm was used for variant and genotype calling [50]. The final VCF file contained 12931 SNP markers that were present in at least 75% of individuals, had a minor allele detected in at least 2 individuals, and gave genotype likelihoods of at least 95%.

#### ADMIXTURE analysis

To assess if the backcrossing procedure resulted in the expected high genome-wide similarity between the *w*AlbB2-F4 strain and the Australian wild type strain, we performed ADMIXTURE analysis that estimates ancestry proportions for each individual [51]. To avoid estimation bias caused by the highly-linked markers, we pruned SNPs so that they are at least 100 kb apart using VCFtools [52], and ran ADMIXTURE analysis with 3803 unlinked SNP markers (distributed across all three *Ae*. *aegypti* chromosomes) while assuming two ancestral populations (K = 2). Specifically, the number of analysed SNP positions was 920, 1521 and 1362 on chromosome 1, 2 and 3, respectively. The median distance between the adjacent SNPs on chromosome 1 was 239 kb, on chromosome 2 was 223 kb, and on chromosome 3 was 218 kb.

### Insecticide resistance bioassays

Each of the three *Ae*. *aegypti* strains (wild type Australia, WB2 and *w*AlbB2-F4) was assessed for insecticide resistance to cypermethrin, alpha-cypermethrin and lambda-cyhalothrin and bifenthrin, the active constituents of commercially available insecticides in Queensland, Australia. Cypermethrin, alpha-cypermethrin and lambda-cyhalothrin were tested using CDC bottle bioassays. These were performed in insecticide coated glass bottles at their diagnostic dose using acetone as a solvent as per CDC guidelines (https://www.cdc.gov/malaria/resources/pdf/fsp/ir_manual/ir_cdc_bioassay_en.pdf). Females of each strain were divided and allocated into 4–7 treatment bottles (insecticide coated) and 1–2 control bottles (acetone only). Knock down was recorded every five min. After 120 min, mosquitoes were transferred to untreated containers, provided with 10% sucrose ad libitum, and maintained for 24 hr to assess recovery. Susceptibility to bifenthrin was tested using the WHO filter paper assay (https://apps.who.int/iris/bitstream/handle/10665/250677/9789241511575-eng.pdf) at the diagnostic dose of 0.025% and mortality scored at 15 min intervals.

Mosquitoes from wild type, WB2, *w*AlbB2-F4 strains were tested for the presence of mutations in the voltage-gated sodium channel protein (VGSC) gene associated with knock down resistance to pyrethroids using Allele-specific quantitative PCR and melting curve analysis (AS-PCR) [53, 54]. These mutations included single nucleotide mutations causing changes from valine (V) to leucine (L) amino acid substitution at locus 410 (V410L), V to isoleucine (I) at locus 1016 (V1016I) and from phenylalanine (F) to cysteine (C) at position 1534 (F1534C) (numbered according to the homologous locus in the VCSC gene in *Musca domestica*). A strain of *Ae*. *aegypti* from Merida, Mexico, with partial expression of kdr resistance phenotypes was used as a positive assay control.

### Statistics

The median relative staining densities of Alexa 488 (*Wolbachia*) to DAPI (DNA) was compared between tissues using the Kruskal-Wallis test and Dunn’s multiple comparison test. Arbovirus infection rates were compared between *w*AlbB2-F4 and WT mosquitoes using Fisher’s Exact test. The arbovirus titres in body, legs and wings and saliva samples of infected mosquitoes were compared between *w*AlbB2-F4 and WT mosquitoes using the Mann-Whitney test. All statistical analyses were performed in Graphpad Prism, version 8 (GraphPad Software Inc, San Diego CA).

## Supporting information

S1 Dataset Datasets are included for Figs 1F, 2B, 3B and 4B. (DOCX)

S2 Dataset Variant Call Format (VCF) file v.4.2 containing SNPs and genotypes at 3803 unlinked loci across all three chromosomes of *Aedes aegypti*. CNS-C denotes Individuals from the parental wild type colony, WB2-C denotes individuals from the parental wB2 colony, and WB2-B denotes individuals from the backcrossed strain wB2F4. These data were used to perform ADMIXTURE analysis.
(TXT)
