# Supplementary material for: Wolbachia wAlbB inhibit dengue and Zika infection in the mosquito Aedes aegypti with an Australian background
Source: PLoS Negl Trop Dis. 2022 Oct 13;16(10):e0010786. doi: 10.1371/journal.pntd.0010786 (PMC9562151; doi:10.1371/journal.pntd.0010786)
Supplement: S1 Dataset — (DOCX) [file pntd.0010786.s001.docx]

**S1 Datasets**

Datasets are included for Figs 1F, 2B, 3B and 4B.

**Staining areas of Alexa488 and DAPI to calculate the relative staining area of *Wolbachia* to DNA (Fig 1F).** Staining areas were quantified using Aperio eSlide Manager and ImageScope Viewer software (Aperio). Analysis regions were created by circumscribing mosquito organs/tissues (midgut, salivary glands, head, ovary, thoracic ganglia, flight muscle and interstitial tissue) from digital micrographs of immunofluorescence-stained mosquito sections. Analysis was performed by dividing the area of Alexa-488 positive pixels by the area of DAPI positive pixels to provide an approximate estimate of the proportion of cells positive for *Wolbachia*. The analysis regions for interstitial tissue were circumscribed by the margins of the abdomen and thorax but excluding any regions covered by other tissues.

| **SampleID** | **Tissue type** | **DAPI Area (mm^2^)** | **Alexa Fluor 488 Area (mm^2^)** | | **488/DAPI** |  |
| --- | --- | --- | --- | --- | --- | --- |
| S1 | Salivary gland | 0.0010 | | 0.0002 | 0.2146 | |
| S2 | Salivary gland | 0.0010 | | 0.0013 | 1.2919 | |
| S3 | Salivary gland | 0.0024 | | 0.0038 | 1.5811 | |
| S4 | Salivary gland | 0.0015 | | 0.0036 | 2.3562 | |
| S5 | Salivary gland | 0.0023 | | 0.0044 | 1.9092 | |
| S6 | Salivary gland | 0.0018 | | 0.0030 | 1.7068 | |
| S7 | Salivary gland | 0.0011 | | 0.0012 | 1.0698 | |
| S8 | Salivary gland | 0.0042 | | 0.0043 | 1.0351 | |
| S9 | Salivary gland | 0.0026 | | 0.0062 | 2.3747 | |
| S10 | Salivary gland | 0.0016 | | 0.0046 | 2.9304 | |
| S11 | Salivary gland | 0.0009 | | 0.0005 | 0.5078 | |
| S12 | Salivary gland | 0.0008 | | 0.0004 | 0.5437 | |
| O1 | Ovary | 0.0270 | | 0.0762 | 2.8254 | |
| O2 | Ovary | 0.0504 | | 0.0443 | 0.8779 | |
| O3 | Ovary | 0.0346 | | 0.0281 | 0.8108 | |
| O4 | Ovary | 0.0077 | | 0.0368 | 4.7550 | |
| O5 | Ovary | 0.0389 | | 0.0102 | 0.2633 | |
| O6 | Ovary | 0.0225 | | 0.0986 | 4.3794 | |
| O7 | Ovary | 0.0222 | | 0.0446 | 2.0090 | |
| O8 | Ovary | 0.0320 | | 0.0261 | 0.8173 | |
| O9 | Ovary | 0.0285 | | 0.0382 | 1.3405 | |
| O10 | Ovary | 0.0205 | | 0.0253 | 1.2328 | |
| O11 | Ovary | 0.0254 | | 0.0283 | 1.1149 | |
| O12 | Ovary | 0.0205 | | 0.0189 | 0.9204 | |
| O13 | Ovary | 0.0223 | | 0.0208 | 0.9358 | |
| O14 | Ovary | 0.0154 | | 0.0178 | 1.1559 | |
| O15 | Ovary | 0.0231 | | 0.0110 | 0.4759 | |
| O16 | Ovary | 0.0191 | | 0.0317 | 1.6576 | |
| O17 | Ovary | 0.0118 | | 0.0470 | 3.9696 | |
| O18 | Ovary | 0.0115 | | 0.0244 | 2.1183 | |
| O19 | Ovary | 0.0293 | | 0.0218 | 0.7442 | |
| O20 | Ovary | 0.0364 | | 0.0274 | 0.7541 | |
| O21 | Ovary | 0.0212 | | 0.0227 | 1.0697 | |
| O22 | Ovary | 0.0126 | | 0.0129 | 1.0214 | |
| O23 | Ovary | 0.0155 | | 0.0220 | 1.4168 | |
| O24 | Ovary | 0.0285 | | 0.0281 | 0.9873 | |
| I1 | Interstitial | 0.0554 | | 0.0360 | 0.6504 | |
| I2 | Interstitial | 0.0558 | | 0.0822 | 1.4744 | |
| I3 | Interstitial | 0.0251 | | 0.0166 | 0.6597 | |
| I4 | Interstitial | 0.0282 | | 0.0341 | 1.2094 | |
| I5 | Interstitial | 0.0472 | | 0.0469 | 0.9926 | |
| I6 | Interstitial | 0.0292 | | 0.0201 | 0.6876 | |
| I7 | Interstitial | 0.0454 | | 0.0593 | 1.3062 | |
| I8 | Interstitial | 0.0271 | | 0.0216 | 0.7984 | |
| I9 | Interstitial | 0.0294 | | 0.0137 | 0.4664 | |
| I10 | Interstitial | 0.0329 | | 0.0206 | 0.6247 | |
| I11 | Interstitial | 0.0288 | | 0.0153 | 0.5308 | |
| I12 | Interstitial | 0.0344 | | 0.0259 | 0.7538 | |
| I13 | Interstitial | 0.0819 | | 0.0610 | 0.7441 | |
| I14 | Interstitial | 0.0421 | | 0.0058 | 0.1381 | |
| I15 | Interstitial | 0.0301 | | 0.0226 | 0.7512 | |
| I16 | Interstitial | 0.0477 | | 0.0584 | 1.2257 | |
| I17 | Interstitial | 0.0500 | | 0.0491 | 0.9829 | |
| I18 | Interstitial | 0.0409 | | 0.0420 | 1.0256 | |
| I19 | Interstitial | 0.0289 | | 0.0244 | 0.8434 | |
| H1 | Head | 0.0252 | | 0.0056 | 0.2227 | |
| H2 | Head | 0.0076 | | 0.0082 | 1.0766 | |
| H3 | Head | 0.0117 | | 0.0058 | 0.4962 | |
| H4 | Head | 0.0067 | | 0.0108 | 1.6190 | |
| H5 | Head | 0.0143 | | 0.0068 | 0.4798 | |
| H6 | Head | 0.0071 | | 0.0043 | 0.6119 | |
| H7 | Head | 0.0173 | | 0.0097 | 0.5593 | |
| H8 | Head | 0.0089 | | 0.0084 | 0.9496 | |
| H9 | Head | 0.0217 | | 0.0033 | 0.1540 | |
| H10 | Head | 0.0185 | | 0.0013 | 0.0709 | |
| H11 | Head | 0.0101 | | 0.0049 | 0.4818 | |
| H12 | Head | 0.0210 | | 0.0012 | 0.0592 | |
| H13 | Head | 0.0145 | | 0.0020 | 0.1407 | |
| H14 | Head | 0.0072 | | 0.0037 | 0.5168 | |
| H15 | Head | 0.0136 | | 0.0034 | 0.2474 | |
| H16 | Head | 0.0169 | | 0.0104 | 0.6137 | |
| H17 | Head | 0.0167 | | 0.0052 | 0.3124 | |
| H18 | Head | 0.0143 | | 0.0092 | 0.6473 | |
| TG1 | Thoracic ganglia | 0.0089 | | 0.0006 | 0.0717 | |
| TG2 | Thoracic ganglia | 0.0072 | | 0.0012 | 0.1656 | |
| TG3 | Thoracic ganglia | 0.0031 | | 0.0011 | 0.3548 | |
| TG4 | Thoracic ganglia | 0.0042 | | 0.0004 | 0.0849 | |
| TG5 | Thoracic ganglia | 0.0032 | | 0.0005 | 0.1568 | |
| TG6 | Thoracic ganglia | 0.0045 | | 0.0010 | 0.2181 | |
| TG7 | Thoracic ganglia | 0.0024 | | 0.0008 | 0.3292 | |
| TG8 | Thoracic ganglia | 0.0048 | | 0.0010 | 0.2146 | |
| TG9 | Thoracic ganglia | 0.0036 | | 0.0002 | 0.0644 | |
| TG10 | Thoracic ganglia | 0.0042 | | 0.0002 | 0.0466 | |
| TG11 | Thoracic ganglia | 0.0057 | | 0.0013 | 0.2205 | |
| TG12 | Thoracic ganglia | 0.0040 | | 0.0003 | 0.0692 | |
| TG13 | Thoracic ganglia | 0.0038 | | 0.0002 | 0.0501 | |
| TG14 | Thoracic ganglia | 0.0046 | | 0.0002 | 0.0505 | |
| TG15 | Thoracic ganglia | 0.0026 | | 0.0005 | 0.1980 | |
| TG16 | Thoracic ganglia | 0.0060 | | 0.0012 | 0.2074 | |
| TG17 | Thoracic ganglia | 0.0045 | | 0.0007 | 0.1516 | |
| TG18 | Thoracic ganglia | 0.0037 | | 0.0008 | 0.2253 | |
| TG19 | Thoracic ganglia | 0.0034 | | 0.0006 | 0.1684 | |
| M1 | Midgut | 0.0208 | | 0.0014 | 0.0667 | |
| M2 | Midgut | 0.0312 | | 0.0030 | 0.0953 | |
| M3 | Midgut | 0.0260 | | 0.0023 | 0.0874 | |
| M4 | Midgut | 0.0417 | | 0.0062 | 0.1499 | |
| M5 | Midgut | 0.0244 | | 0.0007 | 0.0289 | |
| M6 | Midgut | 0.0168 | | 0.0004 | 0.0244 | |
| M7 | Midgut | 0.0231 | | 0.0019 | 0.0819 | |
| M8 | Midgut | 0.0324 | | 0.0009 | 0.0266 | |
| M9 | Midgut | 0.0140 | | 0.0005 | 0.0377 | |
| M10 | Midgut | 0.0116 | | 0.0013 | 0.1121 | |
| M11 | Midgut | 0.0154 | | 0.0014 | 0.0885 | |
| FM1 | Flight muscle | 0.0105 | | 0.0003 | 0.0269 | |
| FM2 | Flight muscle | 0.0226 | | 0.0011 | 0.0504 | |
| FM3 | Flight muscle | 0.0101 | | 0.0006 | 0.0546 | |
| FM4 | Flight muscle | 0.0085 | | 0.0003 | 0.0334 | |
| FM5 | Flight muscle | 0.0081 | | 0.0005 | 0.0626 | |
| FM6 | Flight muscle | 0.0064 | | 0.0003 | 0.0410 | |
| FM7 | Flight muscle | 0.0084 | | 0.0002 | 0.0197 | |
| FM8 | Flight muscle | 0.0134 | | 0.0007 | 0.0511 | |
| FM9 | Flight muscle | 0.0079 | | 0.0006 | 0.0704 | |
| FM10 | Flight muscle | 0.0097 | | 0.0004 | 0.0361 | |
| FM11 | Flight muscle | 0.0088 | | 0.0001 | 0.0120 | |
| FM12 | Flight muscle | 0.0115 | | 0.0003 | 0.0225 | |
| FM13 | Flight muscle | 0.0081 | | 0.0002 | 0.0259 | |
| FM14 | Flight muscle | 0.0081 | | 0.0005 | 0.0651 | |
| FM15 | Flight muscle | 0.0117 | | 0.0001 | 0.0065 | |
| FM16 | Flight muscle | 0.0063 | | 0.0000 | 0.0062 | |
| FM17 | Flight muscle | 0.0084 | | 0.0002 | 0.0273 | |
| FM18 | Flight muscle | 0.0120 | | 0.0005 | 0.0431 | |
| FM19 | Flight muscle | 0.0093 | | 0.0005 | 0.0485 | |
| FM20 | Flight muscle | 0.0047 | | 0.0002 | 0.0403 | |
| FM21 | Flight muscle | 0.0043 | | 0.0010 | 0.2388 | |

**Dengue virus densities within sample (bodies, legs and wings and saliva) from wild type and *w*AlbB2-F4 mosquitoes 14 days after feeding on DENV-2 virus in a blood meal (Fig 2B).**

| MosquitoID | Strain | Body titre (log copies per mosquito) | Legs & wings titre (log copies per mosquito) | Saliva titre (log CCID50/ml) |
| --- | --- | --- | --- | --- |
| 1 | Wild type | 7.50 | 4.89 | - |
| 2 | Wild type | - | - | - |
| 3 | Wild type | 7.74 | 5.73 | - |
| 4 | Wild type | 8.08 | 5.49 | na |
| 5 | Wild type | 8.71 | 5.37 | na |
| 6 | Wild type | 8.04 | 5.61 | - |
| 7 | Wild type | 7.64 | 5.47 | - |
| 8 | Wild type | - | - | - |
| 9 | Wild type | 8.24 | 5.52 | - |
| 10 | Wild type | 4.12 | - | - |
| 11 | Wild type | - | - | - |
| 12 | Wild type | - | - | - |
| 13 | Wild type | 8.44 | 5.05 | - |
| 14 | Wild type | - | - | - |
| 15 | Wild type | 8.85 | - | - |
| 16 | Wild type | 8.51 | - | - |
| 17 | Wild type | 8.06 | 5.33 | - |
| 18 | Wild type | 8.69 | 5.26 | na |
| 19 | Wild type | 8.48 | - | 1.70 |
| 20 | Wild type | 3.74 | 4.82 | - |
| 21 | Wild type | 8.03 | 4.66 | - |
| 22 | Wild type | 8.52 | 5.41 | - |
| 23 | Wild type | 3.94 | - | 2.05 |
| 24 | Wild type | 8.39 | - | na |
| 25 | *w*AlbB2-F4 | - | - | - |
| 26 | *w*AlbB2-F4 | - | - | - |
| 27 | *w*AlbB2-F4 | - | - | - |
| 28 | *w*AlbB2-F4 | - | - | - |
| 29 | *w*AlbB2-F4 | - | - | - |
| 30 | *w*AlbB2-F4 | - | - | - |
| 31 | *w*AlbB2-F4 | - | - | na |
| 32 | *w*AlbB2-F4 | - | - | - |
| 33 | *w*AlbB2-F4 | - | - | - |
| 34 | *w*AlbB2-F4 | - | - | - |
| 35 | *w*AlbB2-F4 | - | - | - |
| 36 | *w*AlbB2-F4 | 7.28 | 3.45 | - |
| 37 | *w*AlbB2-F4 | 4.04 | 2.93 | - |
| 38 | *w*AlbB2-F4 | - | - | - |
| 39 | *w*AlbB2-F4 | - | - | - |
| 40 | *w*AlbB2-F4 | 7.92 | 5.50 | - |
| 41 | *w*AlbB2-F4 | - | - | - |
| 42 | *w*AlbB2-F4 | - | - | - |
| 43 | *w*AlbB2-F4 | 7.12 | 5.14 | na |
| 44 | *w*AlbB2-F4 | - | - | - |
| 45 | *w*AlbB2-F4 | - | - | - |
| 46 | *w*AlbB2-F4 | - | - | - |
| 47 | *w*AlbB2-F4 | - | - | - |
| 48 | *w*AlbB2-F4 | - | - | na |

-, sample uninfected. na, sample not available

**Zika virus densities within sample (bodies, legs and wings and saliva) from wild type and wAlbB2-F4 mosquitoes 14 days after feeding on DENV-2 virus in a blood meal (Fig 3B).**

| MosquitoID | Strain | Body titre (log copies per mosquito) | | | Legs & wings titre (log copies per mosquito) | | | | Saliva titre (log CCID50/ml) | |  |
| --- | --- | --- | --- | --- | --- | --- | --- | --- | --- | --- | --- |
| 1 | Wild type | | | 7.51 | | 6.95 | | | | 2.05 | |
| 2 | Wild type | | | 7.67 | | 7.20 | | | | - | |
| 3 | Wild type | | | 7.55 | | 7.20 | | | | 1.70 | |
| 4 | Wild type | | | 7.49 | | 7.31 | | | | 2.75 | |
| 5 | Wild type | | | 7.74 | | 7.14 | | | | 2.40 | |
| 6 | Wild type | | | 7.38 | | 7.25 | | | | - | |
| 7 | Wild type | | | 7.54 | | 6.75 | | | | - | |
| 8 | Wild type | | | 7.09 | | 6.60 | | | | 2.40 | |
| 9 | Wild type | | | 7.41 | | 6.98 | | | | 3.45 | |
| 10 | Wild type | | | 7.16 | | 6.45 | | | | - | |
| 11 | Wild type | | | 7.29 | | 6.90 | | | | - | |
| 12 | Wild type | | | 7.16 | | 6.58 | | | | - | |
| 13 | Wild type | | | 7.56 | | 7.15 | | | | 2.75 | |
| 14 | Wild type | | | 7.80 | | 6.96 | | | | 2.75 | |
| 15 | Wild type | | | 7.47 | | 6.88 | | | | 2.40 | |
| 16 | Wild type | | | 7.75 | | 6.82 | | | | 3.10 | |
| 17 | Wild type | | | 7.69 | | 6.85 | | | | - | |
| 18 | Wild type | | | 7.54 | | 6.52 | | | | - | |
| 19 | Wild type | | | 7.73 | | 7.02 | | | | 1.70 | |
| 20 | Wild type | | | 7.72 | | 6.88 | | | | 1.70 | |
| 21 | Wild type | | | 7.69 | | 6.99 | | | | 1.70 | |
| 22 | Wild type | | | 7.26 | | na | | | | 3.45 | |
| 23 | Wild type | | | 7.55 | | 6.61 | | | | - | |
| 24 | Wild type | | | 7.64 | | 6.61 | | | | - | |
| 25 | *w*AlbB2-F4 | | | 6.98 | | 6.02 | | | | - | |
| 26 | *w*AlbB2-F4 | | | 7.27 | | 6.38 | | | | - | |
| 27 | *w*AlbB2-F4 | | | 7.60 | | 6.93 | | | | - | |
| 28 | *w*AlbB2-F4 | | | 7.13 | | 7.02 | | | | na | |
| 29 | *w*AlbB2-F4 | | | 5.43 | | 4.29 | | | | - | |
| 30 | *w*AlbB2-F4 | | | 7.36 | | 6.98 | | | | - | |
| 31 | *w*AlbB2-F4 | | | 7.03 | | 7.04 | | | | 2.40 | |
| 32 | *w*AlbB2-F4 | | | 6.88 | | 5.75 | | | | - | |
| 33 | *w*AlbB2-F4 | | | 6.59 | | 5.71 | | | | - | |
| 34 | *w*AlbB2-F4 | | | 6.15 | | 6.20 | | | | - | |
| 35 | *w*AlbB2-F4 | | | 6.82 | | 6.73 | | | | - | |
| 36 | *w*AlbB2-F4 | | | 5.94 | | 4.42 | | | | - | |
| 37 | *w*AlbB2-F4 | | | 6.84 | | 4.48 | | | | 2.50 | |
| 38 | *w*AlbB2-F4 | | | 7.00 | | 6.60 | | | | - | |
| 39 | *w*AlbB2-F4 | | | 5.98 | | 5.79 | | | | - | |
| 40 | *w*AlbB2-F4 | | | 6.58 | | 6.12 | | | | - | |
| 41 | *w*AlbB2-F4 | | | 6.41 | | 2.74 | | | | - | |
| 42 | *w*AlbB2-F4 | | 7.10 | | | | 5.95 | - | | | |
| 43 | *w*AlbB2-F4 | | 7.10 | | | | 4.86 | - | | | |
| 44 | *w*AlbB2-F4 | | 7.24 | | | | 6.48 | - | | | |
| 45 | *w*AlbB2-F4 | | 6.48 | | | | 6.42 | - | | | |
| 46 | *w*AlbB2-F4 | | 6.95 | | | | 6.23 | - | | | |
| 47 | *w*AlbB2-F4 | | 7.15 | | | | 6.51 | - | | | |
| 48 | *w*AlbB2-F4 | | 6.66 | | | | 5.79 | - | | | |

-, sample uninfected. na, sample not available

**Survival of different strains of mosquitoes exposed to common insecticides in insecticide resistance bioassays (Fig 4b).**

|  | **Survival of wild type mosquitoes (percent)** | | | | |
| --- | --- | --- | --- | --- | --- |
| **Time (h)** | **Cypermethrin** | **α-Cypermethrin** | **λ-cyhalothrin** | **Bifenthrin** | **Control** |
| 0 | 100.0 | 100.0 | 100.0 | 100.0 | 100.0 |
| 5 | 100.0 | 100.0 | 70.8 | nt | 100.0 |
| 10 | 59.8 | 91.8 | 56.9 | nt | 100.0 |
| 15 | 15.9 | 75.4 | 18.1 | 49.1 | 100.0 |
| 20 | 4.9 | 10.5 | 0.0 | nt | 100.0 |
| 25 | 0.0 | 1.8 | 0.0 | nt | 100.0 |
| 30 | 0.0 | 0.0 | 0.0 | 0.0 | 100.0 |
| 35 | 0.0 | 0.0 | 0.0 | nt | 100.0 |
| 40 | 0.0 | 0.0 | 0.0 | nt | 100.0 |
| 45 | 0.0 | 0.0 | 0.0 | 0.0 | 100.0 |
| 50 | 0.0 | 0.0 | 0.0 | nt | 100.0 |
| 55 | 0.0 | 0.0 | 0.0 | nt | 100.0 |
| 60 | 0.0 | 0.0 | 0.0 | 0.0 | 100.0 |
| 65 | 0.0 | 0.0 | 0.0 | nt | 100.0 |
| 70 | 0.0 | 0.0 | 0.0 | nt | 100.0 |
| 75 | 0.0 | 0.0 | 0.0 | nt | 100.0 |
| 80 | 0.0 | 0.0 | 0.0 | nt | 100.0 |
| 85 | 0.0 | 0.0 | 0.0 | nt | 100.0 |
| 90 | 0.0 | 0.0 | 0.0 | nt | 100.0 |
| 95 | 0.0 | 0.0 | 0.0 | nt | 100.0 |
| 100 | 0.0 | 0.0 | 0.0 | nt | 100.0 |
| 105 | 0.0 | 0.0 | 0.0 | nt | 100.0 |
| 110 | 0.0 | 0.0 | 0.0 | nt | 100.0 |
| 115 | 0.0 | 0.0 | 0.0 | nt | 100.0 |
| 120 | 0.0 | 0.0 | 0.0 | nt | 100.0 |

nt, not tested

|  | **Survival of *w*AlbB2 mosquitoes (percent)** | | | | |
| --- | --- | --- | --- | --- | --- |
| **Time (h)** | **Cypermethrin** | **α-Cypermethrin** | **λ-Cyhalothrin** | **Bifenthrin** | **Control** |
| 0 | 100.0 | 100.0 | 100.0 | 100.0 | 100.0 |
| 5 | 100.0 | 100.0 | 98.5 | nt | 100.0 |
| 10 | 82.0 | 90.2 | 50.0 | nt | 100.0 |
| 15 | 4.0 | 28.6 | 0.0 | 7.7 | 100.0 |
| 20 | 0.0 | 2.0 | 0.0 | nt | 100.0 |
| 25 | 0.0 | 0.0 | 0.0 | nt | 100.0 |
| 30 | 0.0 | 0.0 | 0.0 | 0.0 | 100.0 |
| 35 | 0.0 | 0.0 | 0.0 | nt | 100.0 |
| 40 | 0.0 | 0.0 | 0.0 | nt | 100.0 |
| 45 | 0.0 | 0.0 | 0.0 | 0.0 | 100.0 |
| 50 | 0.0 | 0.0 | 0.0 | nt | 100.0 |
| 55 | 0.0 | 0.0 | 0.0 | nt | 100.0 |
| 60 | 0.0 | 0.0 | 0.0 | 0.0 | 100.0 |
| 65 | 0.0 | 0.0 | 0.0 | nt | 100.0 |
| 70 | 0.0 | 0.0 | 0.0 | nt | 100.0 |
| 75 | 0.0 | 0.0 | 0.0 | nt | 100.0 |
| 80 | 0.0 | 0.0 | 0.0 | nt | 100.0 |
| 85 | 0.0 | 0.0 | 0.0 | nt | 100.0 |
| 90 | 0.0 | 0.0 | 0.0 | nt | 100.0 |
| 95 | 0.0 | 0.0 | 0.0 | nt | 100.0 |
| 100 | 0.0 | 0.0 | 0.0 | nt | 100.0 |
| 105 | 0.0 | 0.0 | 0.0 | nt | 100.0 |
| 110 | 0.0 | 0.0 | 0.0 | nt | 100.0 |
| 115 | 0.0 | 0.0 | 0.0 | nt | 100.0 |
| 120 | 0.0 | 0.0 | 0.0 | nt | 100.0 |

nt, not tested

|  | **Survival of *w*AlbB2-F4 mosquitoes (percent)** | | | | |
| --- | --- | --- | --- | --- | --- |
| **Time (h)** | **Cypermethrin** | **α-cypermethrin** | **λ-Cyhalothrin** | **Bifenthrin** | **Control** |
| 0 | 100.0 | 100.0 | 100.0 | 100.0 | 100.0 |
| 5 | 97.8 | 97.9 | 90.0 | nt | 100.0 |
| 10 | 83.3 | 97.9 | 75.0 | nt | 100.0 |
| 15 | 3.3 | 39.6 | 0.0 | 56.9 | 100.0 |
| 20 | 0.0 | 8.3 | 0.0 | nt | 100.0 |
| 25 | 0.0 | 0.0 | 0.0 | nt | 100.0 |
| 30 | 0.0 | 0.0 | 0.0 | 0.0 | 100.0 |
| 35 | 0.0 | 0.0 | 0.0 | nt | 100.0 |
| 40 | 0.0 | 0.0 | 0.0 | nt | 100.0 |
| 45 | 0.0 | 0.0 | 0.0 | 0.0 | 100.0 |
| 50 | 0.0 | 0.0 | 0.0 | nt | 100.0 |
| 55 | 0.0 | 0.0 | 0.0 | nt | 100.0 |
| 60 | 0.0 | 0.0 | 0.0 | 0.0 | 100.0 |
| 65 | 0.0 | 0.0 | 0.0 | nt | 100.0 |
| 70 | 0.0 | 0.0 | 0.0 | nt | 100.0 |
| 75 | 0.0 | 0.0 | 0.0 | nt | 100.0 |
| 80 | 0.0 | 0.0 | 0.0 | nt | 100.0 |
| 85 | 0.0 | 0.0 | 0.0 | nt | 100.0 |
| 90 | 0.0 | 0.0 | 0.0 | nt | 100.0 |
| 95 | 0.0 | 0.0 | 0.0 | nt | 100.0 |
| 100 | 0.0 | 0.0 | 0.0 | nt | 100.0 |
| 105 | 0.0 | 0.0 | 0.0 | nt | 100.0 |
| 110 | 0.0 | 0.0 | 0.0 | nt | 100.0 |
| 115 | 0.0 | 0.0 | 0.0 | nt | 100.0 |
| 120 | 0.0 | 0.0 | 0.0 | nt | 100.0 |

nt, not tested
